# Profiles of Sterigmatocystin and Its Metabolites during Traditional Chinese Rice Wine Processing

**DOI:** 10.3390/bios12040212

**Published:** 2022-04-01

**Authors:** Jia Zhang, Liwei Xu, Xinxin Xu, Xiaoling Wu, Hua Kuang, Chuanlai Xu

**Affiliations:** 1 State Key Laboratory of Food Science and Technology, Jiangnan University, Wuxi 214122, China; 7200112107@stu.jiangnan.edu.cn (J.Z.); 7170112080@stu.jiangnan.edu.cn (L.X.); xuxinxin@jiangnan.edu.cn (X.X.); kuangh@jiangnan.edu.cn (H.K.); 2 International Joint Research Laboratory for Biointerface and Biodetection and School of Food Science and Technology, Jiangnan University, Wuxi 214122, China; 3 Collaborative Innovation center of Food Safety and Quality Control in Jiangsu Province, Jiangnan University, Wuxi 214122, China

**Keywords:** mycotoxin, food processing, migration and transformation, metabolites, UPLC–MS/MS

## Abstract

Mycotoxin pollution is widespread in cereal, which greatly threatens food security and human health. In this study, the migration and transformation of sterigmatocystin (STG) mycotoxin during the contaminated rice wine processing was systematically assessed. QuEChERS (Quick, Easy, Cheap, Effective, Rugged, and Safe) coupled with ultrahigh-performance liquid chromatography coupled with tandem mass spectrometry (UPLC−MS/MS) method was firstly established for STG analysis in rice wine. It was found that high levels of rice leaven caused a significant reduction in STG in the fermented rice and wine, which was mainly due to the adsorption of yeast cells and *Rhizopus* biological degradation. However, compared with rice, the levels of STG in separated fermented wine was significantly decreased by 88.6%, possibly attributed to its high log Kow (3.81) and low water solubility (1.44 mg/L). The metabolites of STG (i.e., monohydroxy STG) were identified in rice wine fermentation for the first time. Moreover, STG disturbed the metabolic profile rice wine composition mainly by glycine, serine and threonine metabolism, alanine, aspartate and glutamate metabolism, purine metabolism pathway, particularly with regard to eight amino acids and sixteen lipids. This study elucidated the STG migration and transformation mechanism during the rice wine processing. The finding provided new analytical method for mycotoxin exposure and pollutant in food production, which may support agricultural production and food security.

## 1. Introduction

Food security has been severely affected by plant diseases and pollutants such as pesticides, mycotoxins and heavy metals, particularly mycotoxins, which threaten the precarious food supply of millions of people on the planet [1,2]. Mycotoxins are secondary metabolites produced by several fungal species and are known to frequently contaminate small grain crops in the world [3,4,5], which may pose severe threats to human and animal bodies because of their toxicity [6]. Sterigmatocystin (STG) is a polyketide mycotoxin that is structurally related to aflatoxin B_1_ and produced by *Aspergillus flavus, A*. *parasiticus*, *A. versicolor* and *A. nidulans*; the most common source of STG is *A. versicolor* [7,8]. STG can arise due to fungal infestation at the post-harvesting stage in a range of small grain cereals and grain-based products, including maize, rice, rye, wheat, oats, and barley [8], especially rice and oats [9], the concentration levels ranging from 0 to 83 µg/kg [10,11]. Over the past 10 years, the severe contamination problem in cereal grains caused by mycotoxin has drawn increased attention with regards to food safety [3]. Furthermore, the contamination of mycotoxin in food and environment may cause potential toxic effects in human and livestock, including liver cancer [8]. Like aflatoxin B_1_ (AFB_1_), STG resulted as genotoxic, also able to induce DNA damage and form DNA adducts [8]. However, due to the lack of data related to the occurrence of STG, the EFSA Panel on Contaminants in the Food Chain (CONTAM Panel) were not able to carry out a reliable assessment of human and animal dietary exposure. Therefore, it is essential to collect more data relating to STG in food and animal feed to accomplish the assessment of dietary exposure [8].

Rice (*Oryza sativa L.*) is consumed by more than 50% of the world’s population, functions as a vital source to produce foods for a growing population and forage for livestock, especially in Asian countries. Global rice consumption is predicted to increase 61 Mt to reach 582 Mt by 2020–2029 [12]. Furthermore, rice provides 20% of the world’s dietary energy supply and represents a major source of nutrients due to its daily consumption. Rice is consumed in various forms and can be processed into different foods, including wholegrain flour (brown, milled, or parboiled) and fermented products (e.g., rice wine). Chinese rice wine, also named sweet rice wine, is a traditional fermented food in China. Sweet rice wine has high nutritional value and is rich in amino acids and vitamins [13].

Food processing (e.g., roasting, fermentation, bread and cheese making, milling, heating, or enzymes) can potentially lead to alterations in the levels of STG; the extent of these modifications depends on the type of food involved and the food processing conditions [14,15]. The migration, transformation and degradation of contaminants in food processing, such as mycotoxins, is closely related to its physicochemical properties, especially the melting point, the Octanol-Water Partition Coefficient (Kow), water solubility, and vapor pressure [16,17,18]. STG has a high melting point (246 °C) and is relatively insoluble in water (1.44 mg/L at 25 °C); these characteristics make it stable in the food and surroundings. Veršilovskis et al. [18] reported that STG remained stable during the bread-making process; the levels of STG were determined in 5 of the 29 bread samples analyzed at concentrations ranging from 2.4 to 7.1 µg/kg in Riga, Latvia. Metwally et al. [17] reported high levels of STG (80%) in the curd and much lower levels (20%) in the whey during the cheese making process, thus demonstrating the low solubility of STG in aqueous media.

To our knowledge, only a limited amount of data is available for STG and its metabolites during food processing. However, there are many studies relating to the behavior of mycotoxin during food processing [14,15,18]. Most previous studies were carried out in cereals that were naturally or artificially contaminated. However, the behavior of target mycotoxins in contaminated food is often difficult to predict and characterize. This is because of the wide levels of variation exhibited by mycotoxins, including different derivatives, bonding forms, isomers, and precursors; often, these variations are observed synchronously in grains [15]. Therefore, to investigate the real behavior of target mycotoxins during food processing, free of contamination cereals were spiked with the targeted mycotoxins. Although STG are similar to AFB_1_ in terms of chemical structure, literatures on the conversion profile of STG were scarce in food processing [8], particularly with regards to the process used to make rice wine. However, with the increasing levels of attention targeted to the risk assessment of mycotoxins, the need for data related to the behavior of mycotoxins in food processing has become increasingly urgent. Thus, it is necessary to investigate changes in the levels of STG in cereal-based products that are naturally or artificially infected with *Aspergillus versicolor* during food processing. Additionally, the metabolomic profiles of rice were demonstrated to undergo changes during Chinese rice wine fermentation [13]. Currently, most studies on STG metabolism focus on the transformation in animals [19], but there are few studies on the effects of STG on metabolism in fermentation and other processing technology. Thus, it is essential to investigate whether the level of STG has any effect on the contents of various components based on the metabolic profiles, including organic acids, amino acids, and lipids, which in turn leads to changes in the composition and quality in rice wine during fermentation.

It is worth noting that, the complexity of the rice wine product matrix creates potential difficulties for the analysis of STG levels and its metabolites; thus, researchers have begun to focus on the development of propitious analytical methods [20]. We first performed the analysis of STG levels in rice wine samples using UPLC–MS/MS coupled with the modified QuEChERS (“Quick, Easy, Cheap, Effective, Rugged, and Safe”) method. A non-targeted metabolomics methodology was carried out to identify and compare the metabolic differences between the rice wine products treated with and without STG. However, the main purpose was to enforce the scheme for the evaluation of the fate of STG during the rice wine production and identify the key procedures conducing its possible decrease and to identify the different STG levels’ exposure effect on metabolite profiles during rice wine production. This research enhances our knowledge of the effects of food processing on STG levels. Furthermore, data relating to changes in STG levels during rice wine production might provide further insight into the assessment of chronic dietary risk, as determined by the risk quotients (RQs) method and based on Chinese dietary habits. In addition, it was first observed that the transformation of STG was converted into metabolite (monohydroxy STG) during food fermentation.

## 2. Materials and Methods

### 2.1. Rice Pre-Treatment and Rice Wine Preparation

Rice contaminated with STG were acquired by soaking in an aqueous solution referring to the treatment procedures described in previous studies [15,21].

Spiked sample 1: Rice (2 kg) was individually soaked in STG aqueous solution (2 mg/L) for 8 h in a glass beaker (6 L). Subsequently, the rice was allowed to air dry naturally at room temperature (25 °C) for 84 h in order to restore the original state. The treated rice was stored in a freezer at −20 °C until further use.

Spiked sample 2: Rice (0.5 kg) was individually soaked in STG aqueous solution at six spiked levels (0.3, 0.5, 0.8, 1, 1.5, and 2 mg/L) for 8 h in a glass beaker (2 L). The rice was stirred every 0.5 h to ensure the uniform absorption of STG. Then, the soaked rice was separated from the aqueous solution for the next step as a raw material for rice wine processing.

**Figure 1:**
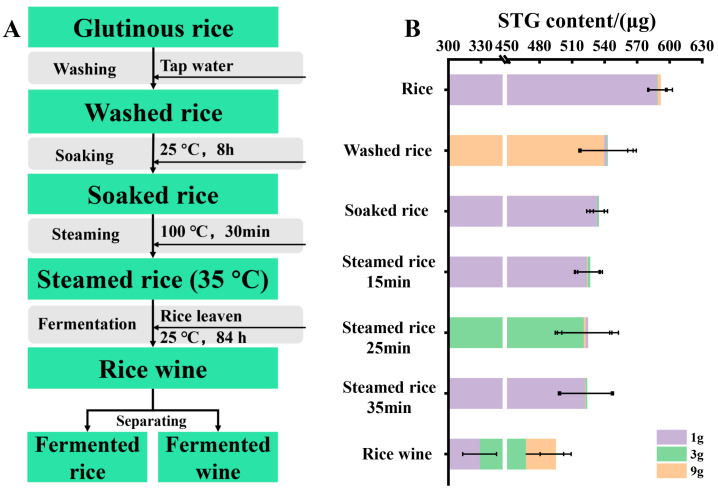
(**A**) The processed step of rice wine. The samples highlighted in green were used for mycotoxin analysis. (**B**) Changes of STG absolute content (μg) in each procedure during rice wine production.

Generally, rice wine processing includes the following consecutive steps, as shown in Figure 1. The treated rice was washed with tap water for 1 min and soaked in water at room temperature (approximately 25 °C) for 8 h. Then, the soaked rice was individually steamed for 30 min. The steamed rice was cooled to about 35 °C and was transferred to a glass (2.5 L). The rice leaven 3 g (ANGEL YEAST CO., LTD., Hubei, China) was dissolved in 100 mL of water; this was then poured into the steamed rice in batches while stirring. Then, the rice was sealed and fermented at room temperature in the dark for 84 h, respectively.

The STG levels of fermented rice and wine were determined separately. The concentration of STG in rice wine was calculated by weighing the content of STG in fermented rice and wine, as follows:

(1) $$C=\sum C1\times \frac{m1}{m1+m2}+C2\times \frac{m2}{m1+m2}$$

In Equation (1), *C* represents the concentration of STG in rice wine (μg/kg), *C*1 represents the content of STG in fermented rice (μg/kg), *C*2 represents the content of STG in fermented wine (μg/kg), *m*1 represents the weight of fermented rice (g), and *m*2 represents the weight of fermented wine (g).

### 2.2. Instrument Conditions

#### 2.2.1. LC–MS/MS Method for STG Analysis

The UPLC System was coupled to a tandem mass spectrometry QTRAP 5500 (AB SCIEX; Toronto, ON, Canada) with electrospray ionization (ESI) in positive mode. This was equipped with a ACQUITY UPLC HSS T3 column (2.1 mm × 100 mm, 1.8 μm; Waters) maintained at 40 °C, and the injection volume was 2 μL. The mobile phase included A: water (0.1% formic acid, 2 mM ammonium formate) and B: acetonitrile. The flow rate was 0.3 mL/min, and the gradient elution is given in Table S1. The mass detection parameters for STG were optimized, including declustering potential (DP), entrance potential (EP), collision energy (CE), and collision cell exit potential (CXP) (Table S1).

#### 2.2.2. LC–HRMS/MS Method for the Identification of STG Degradation Products and Non-Targeted Metabonomics

Chromatographic analysis and the identification of STG metabolites was performed by the Vanquish UHPLC system equipped with a reverse-phase C18 Acquity UPLC HSS T3 column (2.1 mm × 100 mm, 1.8 μm; Waters) heated to 35 °C, coupled with high-resolution tandem mass spectrometer (HRMS/MS) Q-Exactive (Thermo Scientific, Bremen, Germany) equipped with a heated ESI probe. The injection volume was 5 μL and sample determination was carried out over a 22-minute run time. The mass spectrometer was operated in full MS-data-dependent MS/MS (full MS-dd MS/MS) mode [22]. Detailed instrumental and chromatographic conditions are shown in Table S2.

### 2.3. Statistical Analysis

Differences were considered to be statistically significant if *p* < 0.05. Comparisons were carried out by paired-samples *t*-tests using SPSS version 19.0 software. A nonlinear curve fitting equation (Y = *a*X^2^ + *b*X + *c*) was performed using Origin Pro version 9.0 software. The coefficient of determination (R^2^) was used to evaluate the nonlinear curve fitting results and evaluate whether equations had a satisfying goodness of fit and good predictive capability.

The partial least squares–discriminant analysis (PLS–DA) model [23] was accomplished using SIMCA-P (V14.1) for discriminate between different groups. The variable importance in projection (VIP) were calculated by this model. Permutation tests (*n* = 200) were used to evaluate the quality of each PLS–DA model (Figure S4) [24]. Permutation tests were also used to assess whether a particular classification of individuals in either of the designed groups was prominently better than any other random classification in two arbitrary groups. *p* values were calculated by one-way analysis of variance (ANOVA) and pathway analysis was based on metabolites identified by MetaboAnalyst 5.0.

## 3. Results and Discussion

### 3.1. Method Validation

The method validation was assessed according to guidelines and standards from the European Commission [25,26].

The overview of the acquired validation parameters are summarized in Table 1. The product ion chromatograms of STG in fermented rice and wine are shown in Figure S1. The linearity of the calibration curve was evaluated with a standard solution of blank matrix extracts and acetonitrile; this was performed by preparing five matrix-matched calibration standards (5, 10, 20, 100 and 200 μg/L) for STG in each matrix (acetonitrile, soaked rice, steamed rice, fermented rice and fermented wine). Outstanding linearity (R^2^ ≥ 0.9910) was observed in each matrix (Table 1). Matrix effects (ME) were calculated by the slope ratios of the matrix and solvent calibration curves. As shown in Table 1, the results suggested no significant enhancement or suppression effects for STG in soaked rice, steamed rice, and fermented rice, within 10% of the slope ratio, ranging from 0.93 to 1.09. The slope ratio (1.35) of fermented wine showed the matrix enhancement effect. Recovery was performed by spiked experimental samples at three different levels (20, 100, and 200 μg/kg) of STG. Mean recovery of STG ranged from 102–119% for soaked rice, 77–112% for steamed rice, 116–118% for fermented rice, 73–119% for fermented wine, with RSDs ranging from 2.1–8.7% (Table 1). The limits of detection (LODs, signal-to-noise ratio = 3) defined as the minimum detection level, and limits of quantification (LOQs, signal-to-noise ratio = 10) defined as the minimum quantitation level for STG, were 0.01 and 0.03 μg/kg for all matrices (0.07 and 0.25 μg/kg for fermented wine) (Table 1).

### 3.2. The Fate of STG within the Chinese Rice Wine Process

The process used to make traditional Chinese rice wine involves four key steps: washing, soaking, steaming, and fermenting [13]. Although mycotoxins are stable, the levels and structure of STG may change as a result of the complex physicochemical modifications that occur during the processing of raw materials into a processed product [14,27].

#### 3.2.1. Washing and Soaking

The occurrence and concentration of STG in rice wine products are shown in Figure 1, Figure S2 and Table 2. In the present study, the treated rice samples were washed with water for 3 min. During the washing process, the initial level of STG (986.1 μg/kg) in rice had a significant decrease of 16.6% (*p* < 0.05) in STG levels; this may be due to the STG dilution with the water absorption of rice or STG partially dissolved into water during the washing process. Rice could sufficiently absorb amounts of water during soaking, which is conducive to the next effective steaming. Compared to the level of STG in washed rice, 8.7% (*p* < 0.05) of STG was removed by soaking (Figure S2 and Table 1). When washed rice absorbs water and expands, it follows that some STG is redistributed into the soaking water, thus resulting in a decrease of STG in soaked rice.

#### 3.2.2. Influence of Steaming Time on STG Levels

In order to investigate the impact of steaming time on the level of STG during rice wine production, steaming times of 15, 25 and 35 min were used, respectively. The results of different steaming conditions on STG concentration in soaked rice are demonstrated in Table 2 and Figure S2. The levels of STG in soaked rice decreased after steaming (*p* > 0.05) by 1.6%, 2.3%, and 1.7%, after 15, 25, and 35 min, respectively (Table 2). These decreases of STG levels did not differ significantly (*p* > 0.05) when compared across different steaming times (Figure 1 and Figure S2). Our results were in line with the previous studies, veršilovskis et al. [18] found that the levels of STG were stable during bread production (17 min, 200–220 °C); Wu et al. [15] found that the deoxynivalenol (DON) was stable during a Chinese steamed bread making process (20 min, 100 °C). This may be related to the high melting point of STG at 245–246 °C [8].

Steaming is the most widely used method for rice processing and the treatment time is an essential processing factor for food production. Although some studies have reported that different processing times exerted influence on the levels of mycotoxin in food material [15], this is the first detailed investigation reporting changes in the profile of STG in response to different thermal treatments.

#### 3.2.3. Influence of Rice Leaven Levels on the Concentration of STG

**Figure 2:**
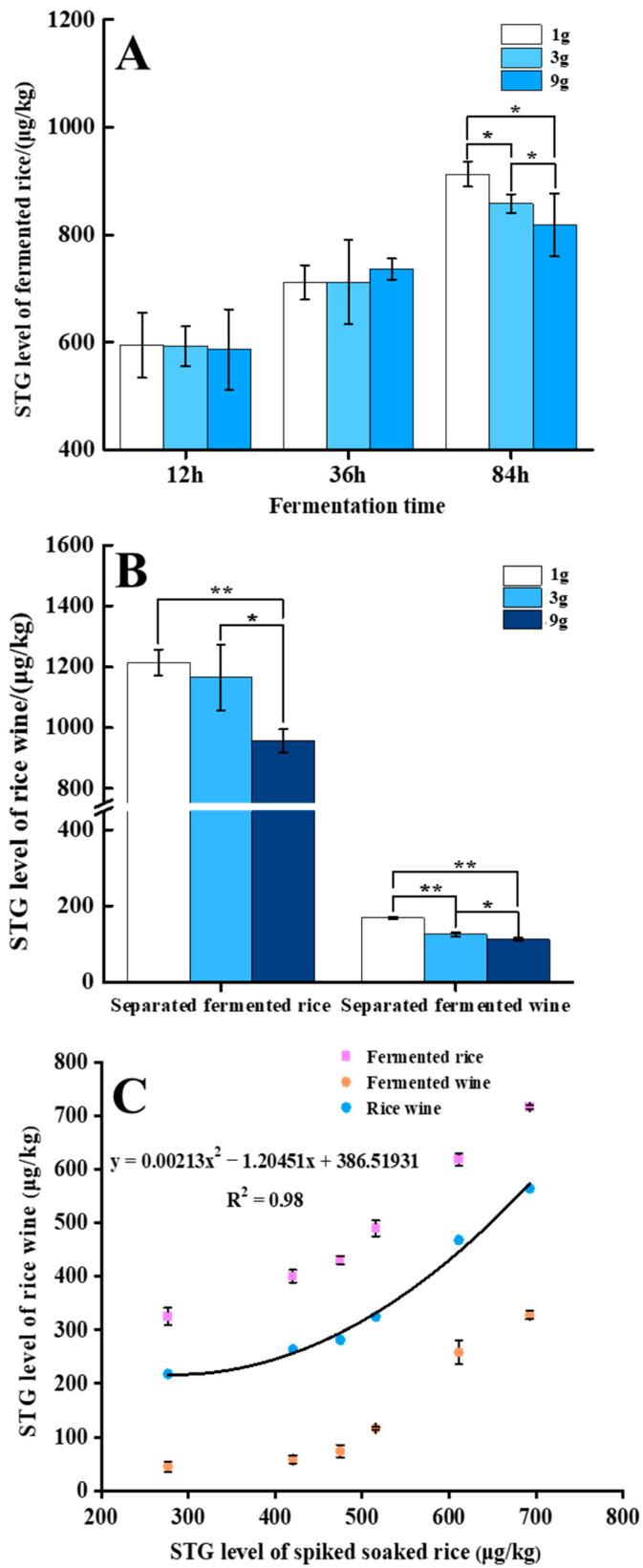
(**A**) The STG level in fermented rice of different fermentation time (12 h, 36 h, 84 h) during rice wine production (1g, 3g, and 9g mean different rice leaven levels); (**B**) the STG level of fermented wine and fermented rice after complete separation; (**C**) correlation of STG level between the original soaked rice and final rice wine product. Data are expressed as means ± standard error of means (*n* = 3). * Error bars represent the standard deviation. * Indicates a significant difference of STG content in rice wine product of the step versus the prior step, (*: *p* < 0.05, **: *p* < 0.01), as determined by Student’s *t*-test.

Prior to the fermentation process, rice leaven was added at low, medium, and high levels (1, 3, and 9 g, respectively). Then, 100 mL of water was added to the steamed rice, mixed thoroughly, and then fermented in a sealed container. As is shown in Table 2 and Figure 2A, the fermentation step (12 h and 36 h) caused no significant change in STG concentrations in fermented rice samples when treated with different levels of rice leaven. However, after 84 h of fermentation, the STG concentration (913.1 μg/kg) in the fermented rice that was mixed with 1 g of rice leaven was higher than that (858.1 μg/kg) in the fermented rice that was mixed with 3 g of rice leaven; the lowest level of STG was found in the fermented rice that was mixed with 9 g of rice leaven (Table 2 and Figure 2A). There were significant (*p* < 0.05) reduction (10.4%) in STG levels when compared between fermented rice samples containing 1 g and 9 g of rice leaven (Figure 2A and Table 2). Therefore, the addition of a larger amount of rice leaven (consisting of yeast and *Rhizopus*) may more easily lead to a reduction in the concentration of STG in rice wine products by binding mycotoxins [28,29]. The relevant study showed lactic acid bacteria and yeast composed of a rather complex biological ecosystem was contributed to dough fermentation [30]. It is possible that this biological ecosystem, containing yeast and *Rhizopus*, plays a key role in the fermentation of rice wine that results in the adsorption, biotransformation, or degradation of STG. Whereas, no other studies have confirmed the prediction. The results of this work were similar to previous studies. Previous research demonstrated that the beta-1,3/1,6-glucan moieties play an essential role during the Saccharomyces cerevisiae cell wall adsorption of the mycotoxin [31]. The glucomannans and mannan-oligosaccharides have been proposed to be the most crucial elements responsible for AFB_1_ binding in yeast [32]. The cell wall of bakery yeast can adsorb 29% of AFB_1_, 68% of zearalenone (ZEA), and 62% of Ochratoxin A (OTA) [33]. Cole et al. found that *Rhizopus* had an effect on biological degradation of the AFB_1_ [34] and Aflatoxin G_1_ (AFG_1_) [35]. Since STG and AFB_1_ are similar in structure, the degradation of STG by *Rhizopus* is similar to that of AFB_1_. Based on the previous literature, and the results of our current research, the weight of rice leaven likely affected the levels of STG via yeast adsorption and *Rhizopus* biological degradation during the rice wine fermentation process. Hence, the addition of high weight rice leaven led to the decrease of STG level.

#### 3.2.4. Influence of Fermentation Time on STG Levels

To monitor the effect of fermentation time on STG levels during rice wine production, three different fermentation times (12, 36, and 84 h) were set as sampling points. The results are shown in Table 2. Compared to the levels of STG in steamed rice, the levels of STG in fermented rice that was mixed with 1 g of rice leaven decreased by 19.4% (12 h) and 3.5% (36 h) and increased by 24% (84 h) of fermentation. The STG level of fermented rice that was mixed with 3 g of rice leaven decreased by 19.6% (12 h) and 3.4% (36 h) and increased by 16.3% (84 h); and the STG levels of fermented rice that was mixed with 9 g of rice leaven decreased by 20.5% (12 h) and 0.1% (36 h) and increased by 11.0% (84 h) (Table 2). Compared to the steamed rice, the decrease in STG in fermented rice at 12 and 36 h was mainly due to the dilution effect of adding water prior to fermentation (Table 2); therefore, after 12 and 36 h of fermentation, the STG concentration had not increased relative to the original STG concentration in steamed rice. As fermentation time increased, the level of STG in fermented rice showed a gradually increasing trend. Generally, the longer the fermentation time, the higher the level of STG in fermented rice (Table 2). Furthermore, the level of STG in separated fermented wine from the three groups supplemented with rice leaven was significantly lower than that in separated fermented rice when fermented rice and fermented wine were completely separated (Table 2). Compared to the level of STG in steamed rice, STG increases of 39.2%, 36.7%, and 22.8% were observed in the separated fermented rice in the 1, 3, and 9g rice leaven groups; STG decreases of 77.1%, 82.9%, and 84.8% were observed in the separated fermented wine in the 1, 3, and 9g rice leaven groups (Table 2). This finding was similar to the results reported by [14], who reported that the levels of fumonisin B_1_ (FB_1_) prominently increased by 166% during the second part of DDGS fermentation in naturally contaminated maize. This is because the rice leaven consisted of complex biological ecosystem [30] converts rice into rice wine via biotransformation during the fermentation process, and STG is a mycotoxin with high fat solubility (log Kow = 3.81) and low water solubility (1.44 mg/L) which tends to be distributed and accumulated in fermented rice [21]. Another reason for the increase in STG level may be yeast extracellular enzymes which might be responsible for STG release from covalent bonds with rice constituents, such as starch or proteins [14].

After the separation of fermented rice and fermented wine, the level of STG in fermented wine were significantly lower than that in fermented rice (Figure 2B and Table 2), this is mainly due to its high log Kow (3.81) and low water-solubility (1.44 mg/L), which caused it to accumulate in fermented rice rather than fermented wine. According to the weight of separated fermented rice and separated fermented wine, the final concentration of STG in rice wine was calculated as 925.4, 850.0 and 613.4 μg/kg, respectively (Table 2). Compared to the STG level in rice, the level of STG decreased by 6.2%, 13.8%, and 37.8% in the final rice wine (1, 3 and 9 g rice leaven, respectively) during rice wine production. Considering the information mentioned previously in Section 3.2.3, it might be assumed that the reduction in STG in the whole rice wine process stage most likely resulted from adsorption by yeasts cells [14,33,36] and *Rhizopus* biological degradation [34,35], or to some extent, via biotransformation.

### 3.3. Correlation of STG Levels between Soaked Rice and Rice Wine Final Product

Fermented wine was generated from fermented rice during fermentation; the fermented rice and fermented wine represented two food matrices with entirely disparate physicochemical properties.

The levels of STG in fermented wine were significantly (*p* < 0.05) lower than the levels in fermented rice. The final levels of STG in rice wine were calculated by Equation (1) and depended on the STG levels in fermented rice and fermented wine (Table S4). A linear relationship was built using the STG levels in the soaked rice and the rice wine products, with a non-linear fitting curve of Y = 0.00213x^2^ − 1.20451x + 386.51931, R^2^ = 0.98 (Figure 2C).

The developed mathematical model demonstrated a satisfying goodness-of-fit and prediction at concentrations ranging from 280 to 700 μg/kg (Figure S3) and showed that the STG level in the final product (such as rice wine) could be evaluated according to the original concentration in raw food material (the STG levels in rice or soaked rice, for instance). Based on the established model, the linear relationship between STG residue levels in rice wine products and rice or soaked rice were also predicted. This clearly demonstrated the potential of this approach to investigate other behaviors of other toxins in the residue of rice wine products and other processed food products.

### 3.4. Identification of Fermentation Degradation Products of STG

**Figure 3:**
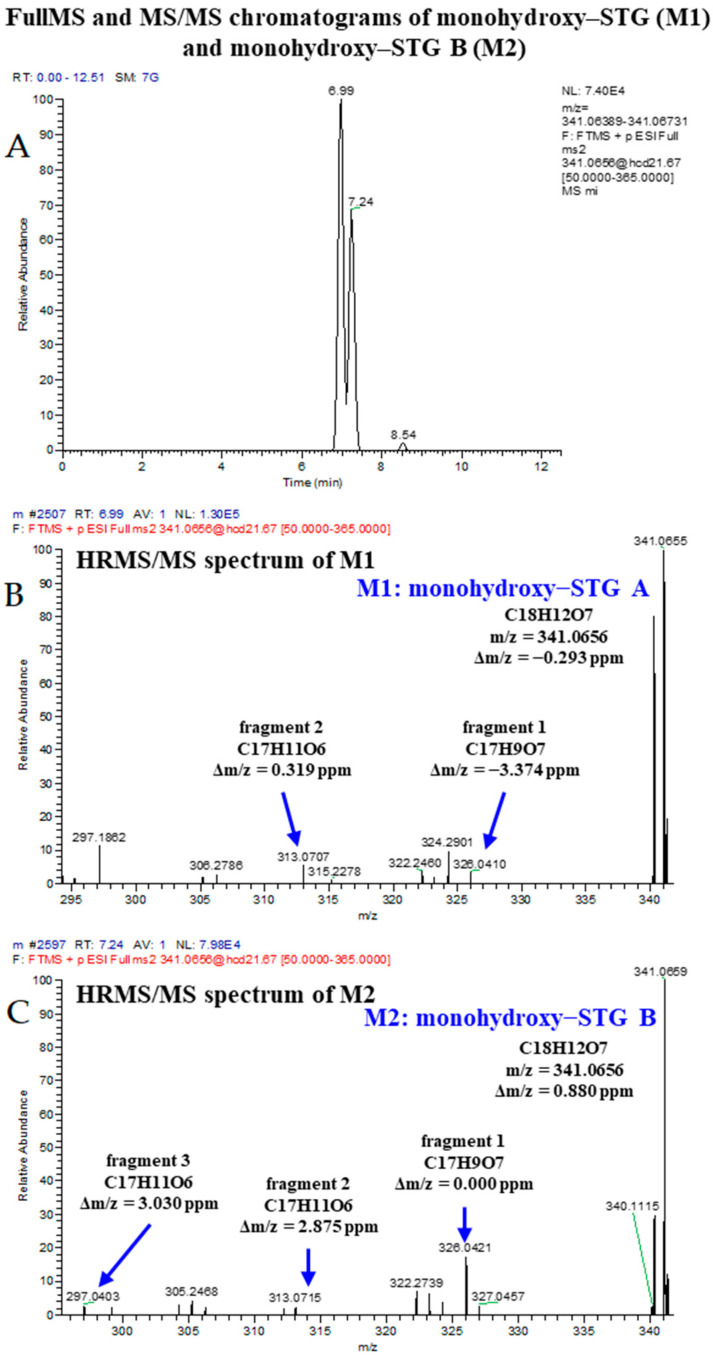
(**A**) Chromatograms of metabolite of STG found in rice wine product; (**B**,**C**) HRMS/MS spectra of metabolites of STG found in rice wine product.

The calculated data clearly showed that the fermentation procedure (9 g) had a noteworthy effect on the final levels of STG in rice wine. There was a 37.8% decrease in the level of STG during the rice wine production (Table 2). In order to investigate whether the changes in STG level was associated with biotransformation products generated by the catalysis of yeast enzymes during the fermentation process. While quantifying STG levels in rice wine products, the biotransformation products of STG were determined by UPLC−HRMS/MS. Two STG biotransformation products, monohydroxy STG A (M1) and monohydroxy STG B (M2), were identified for the first time in the food processing field (Figure 3). The result showed the transformation behavior of STG existed in food processing. The monohydroxy of STG were major metabolite (phase I metabolism) formed by human and rat hepatic microsomes, via hydroxylation of the aromatic ring [8]. Previous studies showed that phase I metabolism of STG consists of a cytochrome P450 (CYP)-mediated formation of mono-hydroxylation reactions [8]. Saccharomyces cerevisiae also contains the P450 enzyme system, which could be the reason of the transformation of STG during fermentation. Unfortunately, it has not been able to quantify for it because there are no standards.

### 3.5. Effects of STG on the Metabolite Profiles of Rice Wine

**Figure 4:**
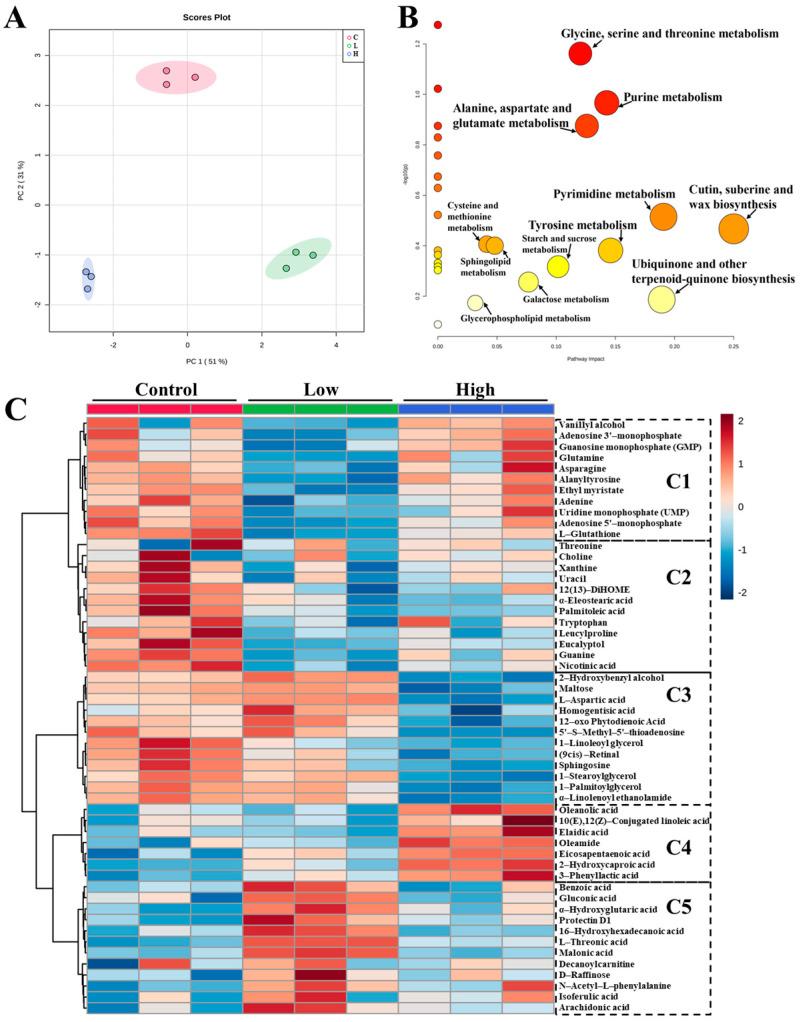
(**A**) PLS-DA score plot of STG exposure rice wine in positive and negative mode results (C, L, and H mean control group, low, and high level STG group); (**B**) pathway impact analysis showing changing metabolism in rice wine treated with STG compared to normal rice wine; (**C**) based on UPLC–HRMS/MS system identified, a heat map of identified metabolites in rice wine with varied STG levels exposure by hierarchical clustering of the most significantly differential metabolites in rice wine (*p* < 0.05 and VIP > 1.0).

Changes in the metabolite profiles during the preparation of rice wine using different groups of rice (with low and high levels of STG) were analyzed by UPLC–HRMS/MS. Partial least-squares discriminant analysis (PLS-DA) of nontargeted metabolites in rice wine samples are shown in Figure 4A. These data demonstrated the quality parameters of the loading plot with an appropriate explanation, including goodness of fit (R2Y), and accuracy (Q2). The permutation test (*n* = 200) was performed on all samples; this confirmed that the model was good quality and that there was no overfitting (Figure S4) [37]. UPLC–HRMS analysis identified a total of 54 highly differential metabolites with a VIP > 1.0 and *p* < 0.05 (Table S5). The PLS-DA models acquired from HRMS analysis showed that the metabolites varied depending on the addition of STG, and each group was obviously discriminated (Figure 4A).

To visualize the changes in metabolite levels based on the STG addition level, a heat map showed that 35 metabolites (Figure 4: C1, C2, and C3) were significantly down-regulated and 19 metabolites (Figure 4: C4, and C5) were significantly up-regulated in a dose-dependent manner when compared with the controls.

Generally, the breakdown of macronutrients can produce micronutrients, such as monosaccharides, amino acids, and lipid metabolites, which can then partake in glycolysis and the TCA cycle to make energy. All metabolites were classified into five groups (C1, C2, C3, C4, and C5) (Figure 4C). Group C1, mainly included amino acids and nucleotides; these decreased when exposed to STG. When compared to the control group, other metabolites in group C2 were also significantly decreased at low and high STG levels. These metabolites were mainly amino acids, nucleic acids, and lipids (Table S5 and Figure 4C). Two saturated lipid acids (α-eleostearic acid and palmitoleic acid) and one oxylipin (DiHOME) were significantly reduced after exposure to STG; these were down-regulated in the low and high STG treatment groups, thus indicating abnormal lipid metabolism. In group C3, these metabolites were significantly reduced in the high STG treatment groups. Although the flavor of long-chain saturated fatty acids is generally regarded as unpleasant, some of them were involved in esterification to form esters during fermentation and aging [13]. Three esters of long-chain saturated fatty acids (1-linoleoyl glycerol, 1-stearoylglycerol, and 1-palmitoylglycerol) were significantly down-regulated in the high STG treatment groups. The metabolites in group C4, including 4 lipids (10(E),12(Z)-Conjugated linoleic acid, Elaidic acid, Oleamide, Eicosapentaenoic acid) and 3 organic acids (Oleanolic acid, 2-Hydroxycaproic acid, 3-Phenyllactic acid) (Table S5 and Figure 4C), were significantly increased after exposure to high levels of STG. Group C5 mainly included six organic acids and others (Table S5 and Figure 4C) which showed a significant increase after exposure to low levels of STG. The distinct changes of these biomarkers revealed a clear metabolite profile for rice wine, and clear indicators of promotion or suppression in the relevant metabolism pathways (Figure 4B). As shown in Figure 4B, the STG exposure may have a negative effect on secretion of yeast protease, lipase and other relevant enzyme using for the hydrolysis of sugars and the breakdown of substances such as proteins and fats [38], which resulted in twelve differential metabolic pathways of rice wine production, especially for 6 pathways of glycine, serine and threonine metabolism, alanine, aspartate and glutamate metabolism, purine metabolism, pyrimidine metabolism, tyrosine metabolism and cutin, suberine and wax biosynthesis. As shown in Figure 4C, the relative contents of amino acids and saturated fatty acids showed a downward trend, while organic acids showed an upward trend, which had a certain influence on the quality of rice wine.

## 4. Conclusions

In this research, we used UPLC–MS/MS to investigate changes in STG levels, and its metabolites, during rice wine production. We found that the levels of STG first decreased but then increased during processing. When compared to the prior initial processing, the levels of STG decreased after washing, soaking, and steaming treatment. High levels of rice leaven may also cause STG levels of fermented rice to fall during fermentation. However, the opposite trend was observed after different fermentation times. Our analysis showed that rice leaven levels and fermentation times were both critical factors in food production. To the best of our knowledge, this study is the first to demonstrate that rice leaven levels and fermentation times can have significant (*p* < 0.05) effects on the levels of STG during rice wine production. Furthermore, for the first time, we also identified the presence of metabolites of STG (monohydroxy STG) during food processing. We also investigated relative changes in the characteristic metabolism of amino acids, lipids, and organic acids. This analysis showed that metabolite profiles changed due to exposure to STG during rice wine fermentation. In conclusion, the results of this study provide a preliminary investigation of STG residues behavior and exposure effect on composition of rice wine during fermentation production and constitute a reference for formulating future rice wine process and risk-assessment programs. In addition, more studies would be essential to replenish the conditions of STG during food production.

## Figures and Tables

**Table 1:** Linear range (μg/L), regression equation, calibration curve coefficients (R^2^), Matrix effects (ME), Limit of detection (LOD) and Limit of quantitation (LOQ) for STG in rice wine products. Recoveries and RSDs of sterigmatocystin in rice wine products at different spiked levels (*n* = 5).

Mycotoxin	Matrix	Linear Range	Regression Equation	R^2^	ME/%	LOD (μg/kg)	LOQ (μg/kg)
STG	Solvent	5–200	y = 190,442x + 268,234	0.9953			
	Soaked rice	5–200	y = 176,167x + 236,281	0.9979	−7.0	0.01	0.03
	Steamed rice	5–200	y = 179,674x + 238,395	0.9969	−6.0	0.01	0.03
	Fermented rice	5–200	y = 208,146x + 392,325	0.9959	+9.0	0.01	0.03
	Fermented wine	5–200	y = 256,280x + 565,254	0.9910	+35.0	0.07	0.25
Sample	20 μg/kg		100 μg/kg		200 μg/kg		
	Recoveries (%)	RSD (%)	Recoveries (%)	RSD (%)	Recoveries (%)		RSD (%)
Soaked rice	102	2.3	107	2.3	119		2.4
Steamed rice	77	6.2	85	8.7	112		3.5
Fermented rice	118	2.1	118	7.6	116		6.9
Fermented wine	73	3.6	105	5.0	119		4.3

**Table 2:** Changes of STG level in spiked samples in different steam time, fermentation time and rice leaven addition level during the rice wine production (mean, *n* = 3).

Sample	Rice	Washed Rice	Soaked Rice	Steam Rice			Fermented Rice-1 g			Fermented Rice-3 g		
				15 min	25 min	35 min	12 h	36 h	84 h	12 h	36 h	84 h
Level/(μg/kg)	986.1	822.3 *	750.8 *	738.8 ^a^	733.2 ^a^	737.7 ^a^	594.8 *^,a^	711.6 *^,b^	913.1 *^,c^	592.8 *^,a^	712.3 *^,b^	858.1 *^,c^
SD	16.4	39.8	14.4	16.2	46.4	37.1	60.8	32.3	23.6	36.8	78.4	17.7
Sample	Fermented rice-9g			Separated fermented wine			Separated fermented rice			Total rice wine		
	12 h	36 h	84 h	1 g	3 g	9 g	1 g	3 g	9 g	1 g	3 g	9 g
Level/(μg/kg)	586.6 *^,a^	736.7 *^,b^	818.5 *^,c^	169 ^a^	126.2 ^b^	112.2 ^c^	1214.1 ^a^	1164.9 ^a^	956.0 ^b^	925.4	850.6	613.4 *
SD	74.9	19.4	58.3	2	5.5	3.9	43.1	108.4	39.0			

Note: * Indicates a significant difference of STG in rice wine product of the step versus the prior step (*p* < 0.05), as determined by Student’s *t*-test. ^a,b,c^ The different letters show a remarkable difference (*p* < 0.05) between the effects of the different factors in same processing; conversely, the same letter shows no significant difference observed. Fermented rice-1 g: the 1 g level of rice leaven during rice wine production, all else follows.

## Data Availability

Not applicable.

## Supplementary Materials

The following supporting information can be downloaded at: https://www.mdpi.com/article/10.3390/bios12040212/s1, Figure S1: TIC of STG in fermented rice (A1) and wine (B1); product ion chromatograms of STG in fermented rice (A2, A3) and wine (B2, B3). Figure S2. Changes of STG level in fermented rice during rice wine production. Figure S3. Changes of STG level in spiked samples during the rice wine process. Note: Group 1–6, spiking STG levels of 276.7, 420.3, 474.9, 515.7, 611.2, 692.7 μg/kg, respectively (Table S4). Data are expressed as means ± standard error of means (*n* = 3). Error bars represent the standard deviation. * Indicates a significant difference of STG in rice wine product of the step versus the prior step (* *p* < 0.05, *** *p* < 0.001), as determined by Student’s t-test. Figure S4. Permutation test on fermented rice (FR) and fermented wine (FW) of exposure groups to control group on PLS-DA model. Spectra are randomly assigned to a class by 200 permutations. (D1, D3, E1, E3) Low level treatment; (D2, D4, E2, E4) High level treatment. Table S1. Chromatography gradient elution procedure and mass parameters Table S2. Instrumental and chromatographic conditions for the analysis of metabolomics in rice wine samples. Table S3. Changes of STG absolute content (μg) in each procedure during rice wine production (mean ± SD, *n* = 3). Table S4. Changes of STG level in spiked samples during rice wine production (mean ± SD, *n* = 3). Table S5. Differential metabolites of rice wine by untargeted metabolomics (*p* < 0.05 and VIP > 1).

## Abbreviations

UPLC–MS/MS, ultra-performance liquid chromatography coupled with tandem mass spectrometry; HRMS, high resolution mass spectrometer; Kow, Octanol-Water Partition Coefficient; Sw, solubility water; STG, sterigmatocystin; DON, deoxynivalenol; AFB_1_, Aflatoxin B_1_; AFG_1_, Aflatoxin G_1_; QuEChERS (Quick, Easy, Cheap, Effective, Rugged, and Safe); ZEA, zearalenone. DDGS, distiller’s dried grains with solubles; OTA, Ochratoxin A.
