# Supplementary material for: Profiles of Sterigmatocystin and Its Metabolites during Traditional Chinese Rice Wine Processing
Source: Biosensors (Basel). 2022 Apr 1;12(4):212. doi: 10.3390/bios12040212 (PMC9028121; doi:10.3390/bios12040212)
Supplement: Supplementary file 1 [file biosensors-12-00212-s001.zip › biosensors-1648422-supplementary.pdf]

biosensors

MDPI

Supporting Information

Profiles of Sterigmatocystin and Its Metabolites during Traditional Chinese Rice Wine Processing

Jia Zhang [1,2,3], Liwei Xu [1,2,3], Xinxin Xu [1,2,3], Xiaoling Wu [1,2,3,*], Hua Kuang [1,2,3] and Chuanlai Xu [1,2,3,*]

1 State Key Laboratory of Food Science and Technology, Jiangnan University, Wuxi 214122, China; 7200112107@stu.jiangnan.edu.cn (J.Z.); 7170112080@stu.jiangnan.edu.cn (L.X.); xuxinxin@jiangnan.edu.cn (X.X.); kuangh@jiangnan.edu.cn (H.K.)
2 International Joint Research Laboratory for Biointerface and Biodetection and School of Food Science and Technology, Jiangnan University, Wuxi 214122, China
3 Collaborative Innovation center of Food Safety and Quality Control in Jiangsu Province, Jiangnan University, Wuxi 214122, China
* Correspondence: wuxiaoling@jiangnan.edu.cn (X.W.); xcl@jiangnan.edu.cn (C.X.); Tel./Fax: +86-510-85329077 (X.W.; C.X.)

1. Chemicals and reagents

Analytical standard sterigmatocystin (STG) was purchased from Dr. Ehrenstorfer GmbH (Augsburg, Germany). The standard stock solution (1 mg/mL) for STG was dissolved in acetonitrile and a working standard solution was diluted into a standard solution of 10 mg/L. All solutions were stored at -20 °C until use. The HPLC-grade acetonitrile/Methanol and HPLC-grade formic acid used for sample extraction were obtained from ANPEL Laboratory Technologies (Shanghai, China). Analytical-grade anhydrous magnesium sulfate ($MgSO_4$) and sodium chloride (NaCl) were purchased from Beijing Chemical and Reagent (Beijing, China). Ultra-pure water was used in all experiments (<18 MU cm resistivity). Primary secondary amine (PSA, 40–63 μm) was obtained from ANPEL Laboratory Technologies (Shanghai, China). LC-MS grade acetonitrile and formic acid were acquired from ANPEL Laboratory Technologies (Shanghai, China).

2. Sample preparation

2.1. Extraction and purification of STG in rice, steamed rice, fermented rice and wine samples

A total of 5 g of each sample was homogenized and weighed into a 50 mL PTFE centrifuge tube, extracted with 2.5 mL of water (fermented wine and steamed rice samples extracted with 0 and 10 mL of water, respectively) and 10 mL of acetonitrile for 2 min using a Vortex Genie 2T (SI, USA) at 1,200 strokes/min. Then, NaCl (2g) and anhydrous $MgSO_4$ (2g) (steamed rice added with 6g NaCl and anhydrous 6g $MgSO_4$) were added and vortexed for 1 min. Samples were then centrifuged for 5 min at 5,000 rpm, and then 1 mL of the supernatant was transferred into a 2 mL centrifuge tube containing 20 mg of PSA sorbents and 150 mg of anhydrous $MgSO_4$. Next, the sample was vortexed vigorously for 30 s and then centrifuged at 12,000 rpm for 1 min. The supernatant was then filtered with a 0.22 μm filter prior to LC-MS/MS.

2.2. Sample preparation for the non-targeted metabonomic analysis of rice wine

Samples were prepared in accordance with a previously described method but with some modifications (Magnuson et al., 2020; Xu et al., 2020). In brief, 200 milligrams of each rice wine sample were homogenized (20 μL of internal standard of DL-2-chlorophenylalanine, 500.0 mg/L) with 800 μL of water (except for fermented wine samples) in 5 mL centrifuge tube, and then 3.2 mL of methanol/ACN (1:1, v/v) was added. The samples were vortexed for 30 s and then extracted ultrasonically for 5 min. The samples were then vortexed for 30 s and centrifuged at 12,000 rpm at 4 °C for 15 min. The supernatants were evaporated to dryness at 4 °C, reconstituted with 200 μL of methanol/ACN/H_2O (4:4:2,

v/v/v), and then vortexed and centrifuged, as described above. Then, 120 μL of supernatant was analyzed by UPLC-HRMS (high resolution mass spectrometer). Quality control (QC) samples were prepared with 20 μL of each sample.

Magnuson, J. T., Giroux, M., Cryder, Z., Gan, J., & Schlenk, D. (2020). The use of non-targeted metabolomics to assess the toxicity of bifenthrin to juvenile chinook salmon (oncorhynchus tshawytscha). *Aquatic toxicology, 224*, 105518.

Xu, L. W., Guo, L. L., Wang, Z. X., Wu, X. L., Kuang, H., Xu, C. L., et al. (2020). Profiling and identification of biocatalyzed transformation of sulfoxaflor in vivo. *Angewandte Chemie International Edition, 59*, 16218–16224.

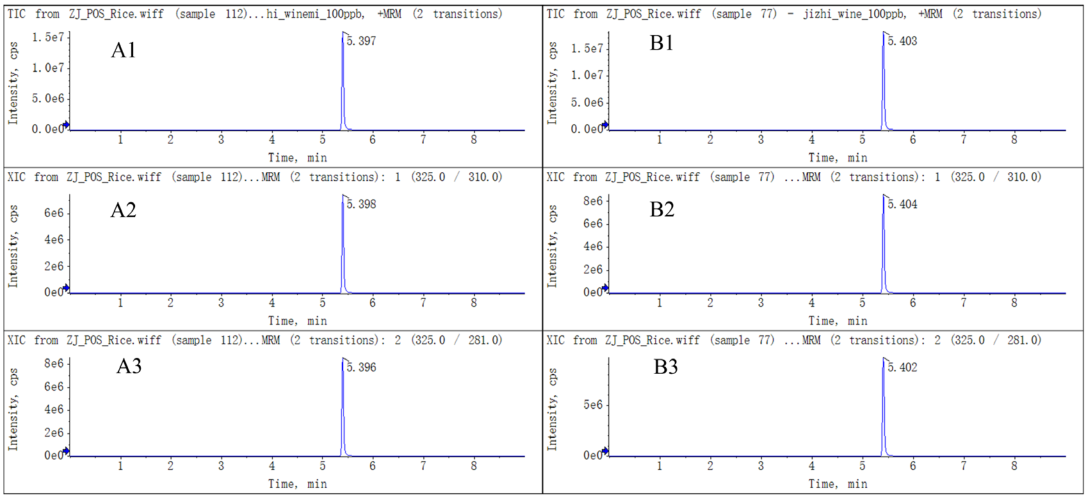

Figure S1. TIC of STG in fermented rice (A1) and wine (B1); product ion chromatograms of STG in fermented rice (A2, A3) and wine (B2, B3).

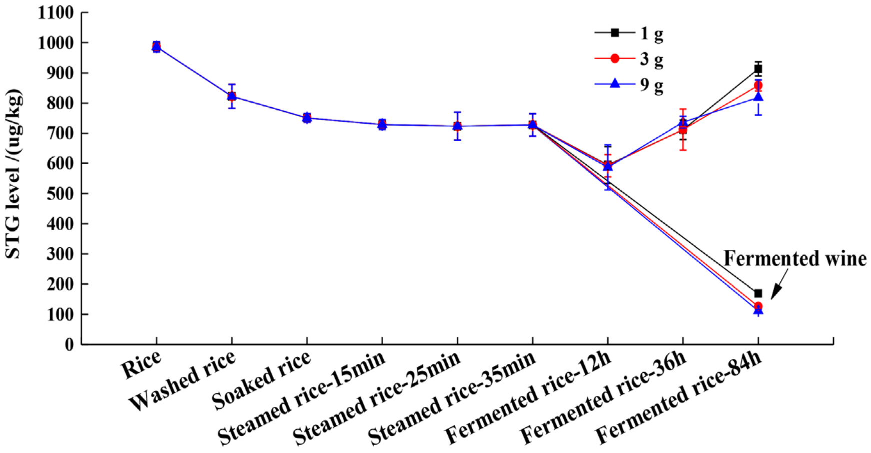

Figure S2. Changes of STG level in fermented rice during rice wine production.

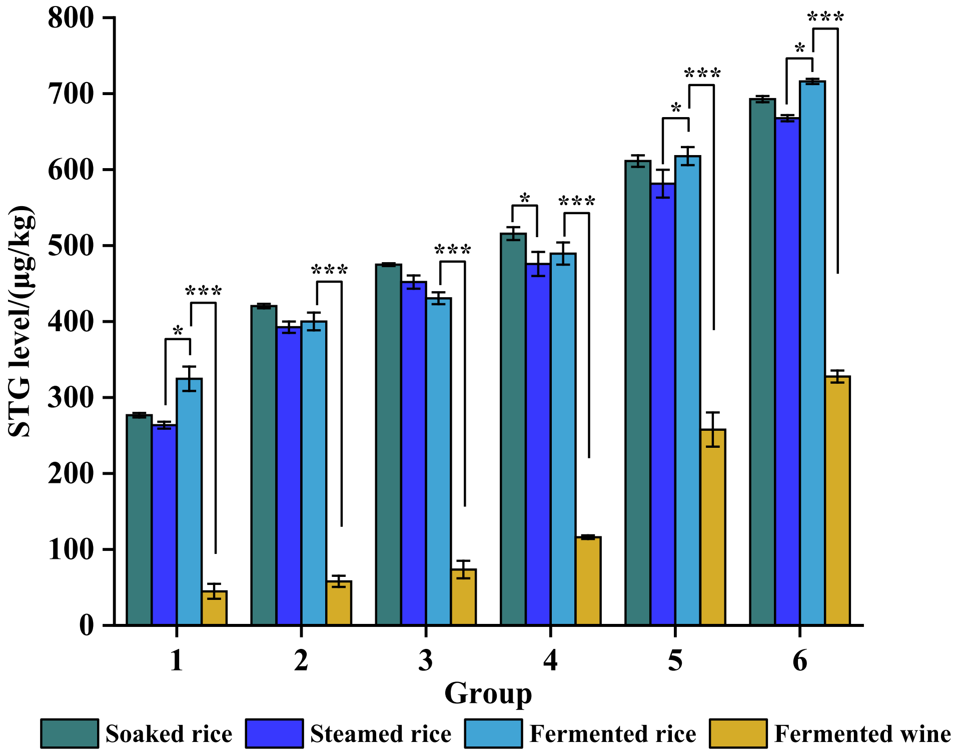

Figure S3. Changes of STG level in spiked samples during the rice wine process. Note: Group 1–6, spiking STG levels of 276.7, 420.3, 474.9, 515.7, 611.2, 692.7 μg/kg, respectively (Table S4**)**. Data are expressed as means ± standard error of means (n = 3). Error bars represent the standard deviation. * Indicates a significant difference of STG in rice wine product of the step versus the prior step (**p* < 0.05, ****p* < 0.001), as determined by Student's t-test.

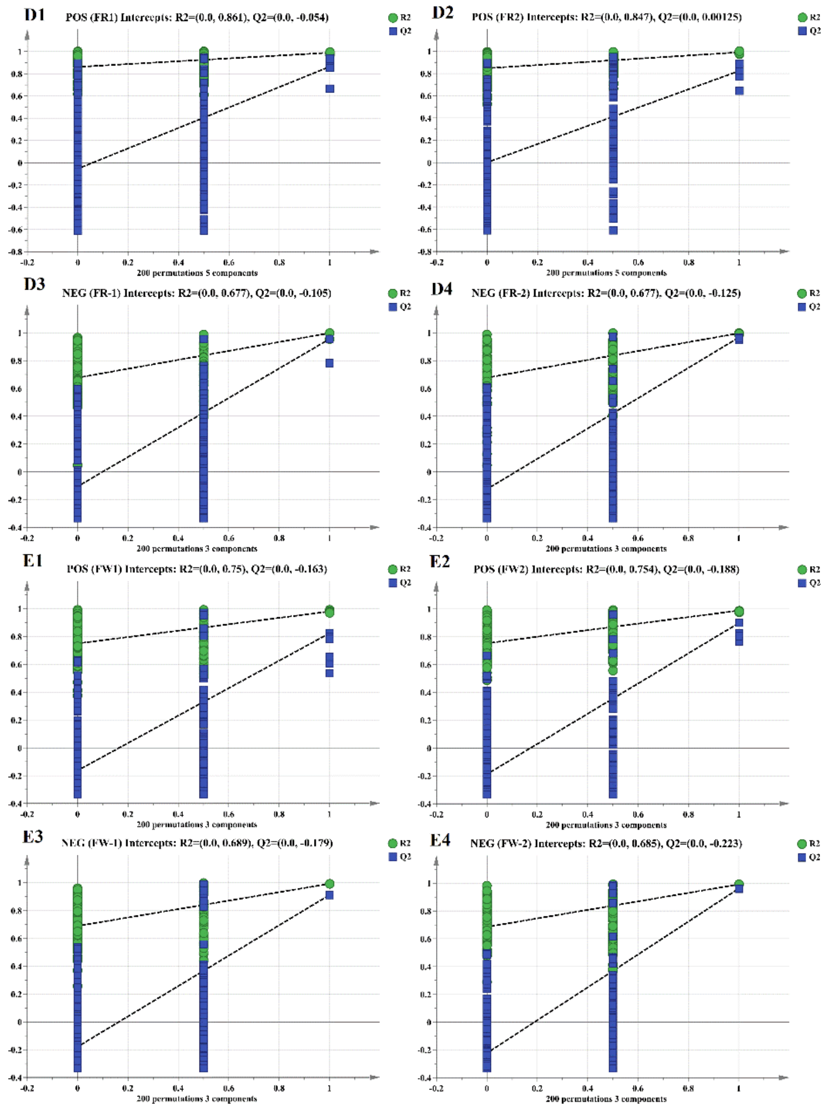

Figure S4. Permutation test on fermented rice (FR) and fermented wine (FW) of exposure groups to control group on PLS-DA model. Spectra are randomly assigned to a class by 200 permutations. (D1, D3, E1, E3) Low level treatment; (D2, D4, E2, E4) High level treatment.

Table S1. Chromatography gradient elution procedure and mass parameters for STG analysis by LC-MS/MS.

Mobile Phase	A: water (0.1% formic acid, 2mM ammonium formate)<br>B: acetonitrile			
Gradient Profile	Time/min	A (%)	B (%)	Flow rate/mL·min^{-1}
	0	55	45	0.3
	1	55	45	0.3
	10	10	90	0.3
	10.1	55	45	0.3
	11.5	55	45	0.3
Mass Parameters	Curtain gas: 35 psi<br>Ion source gas 1 and gas 2: 60 psi<br>Source Temperature: 550 °C<br>Ionspray voltage: 5.5 kV			

Table S2. Instrumental and chromatographic conditions for the analysis of metabolomics in rice wine samples.

Mobile Phase	Positive mode<br>A: water (0.1% formic acid)<br>B: acetonitrile			
	Negative mode<br>A: water (0.1% formic acid+2 mM ammonium acetate)<br>B: acetonitrile			
Gradient Profile	Time/min	A (%)	B (%)	Flow rate/mL·min^{-1}
	0	95	5	0.35
	1.5	95	5	0.35
	15	0	100	0.35
	17	0	100	0.35
	17.1	95	5	0.35
	22	95	5	0.35
Mass Parameters	Scan range (m/z) = 70 to 1050<br>Collision energy (eV) = 20, 40, 60<br>Capillary temperature (℃) = 320			

Table S3. Changes of STG absolute content (μg) in each procedure during rice wine production (mean ± SD, n = 3).

Sample	Rice	Washed rice	Soaked rice	Steam rice			Rice wine-1g	Rice wine-3g	Rice wine-9g
				15min	25min	35min			
Content/(μg)	591.7	542.7*	533.1	524.5^{a}	520.6^{a}	523.8^{a}	494.8^{b}	467.2^{b}	328.7^{a}
SD	10.9	26.5	9.6	11.4	32.1	24.4	14.3	35.0	15.6

Note: * Indicates a significant difference of STG in rice wine product of the step versus the prior step (p < 0.05). The different letters show a remarkable difference (p < 0.05) between the effects of the different factors in same processing; conversely, the same letter shows no significant difference observed, as determined by Student's t-test. Rice wine-1g: the 1g level of rice leaven during rice wine production, all else follows.

Table S4. Changes of STG level in spiked samples during rice wine production (mean ± SD, n = 3).

**No.**	**Concentration**			
	**Soaked rice**	**Steamed rice**	**Fermented rice**	**Fermented wine**
1	276.7 ± 2.9	263.6 ± 4.6	324.7* ± 16.0	44.9*** ± 9.8
2	420.3 ± 2.9	392.6 ± 7.5	400.1 ± 11.7	58.0*** ± 7.3
3	474.9 ± 1.9	452.0 ± 8.7	430.6 ± 7.8	73.5*** ± 11.5
4	515.7 ± 8.5	475.8 ± 15.9*	489.4 ± 14.6	116.1*** ± 2.4
5	611.2 ± 7.5	581.5 ± 18.3	617.7* ± 11.9	257.8*** ± 22.5
6	692.7 ± 4.1	667.7 ± 4.1	716.1* ± 3.4	327.6*** ± 7.9

* Indicates a significant difference of STG in rice wine product of the step versus the prior step (*p < 0.05, *** p < 0.001), as determined by Student's t-test.
Note: Soaked rice samples 1–6, total 6 STG spiking levels (μg/kg).

Table S5. Differential metabolites of rice wine by untargeted metabolomics ($p < 0.05$ and VIP > 1).

No.	HMDB ID	Compound name	Formula	VIP value	*P* value
1	HMDB0004705	12(13)-DiHOME	C18H34O4	1.063	5.69×10^{-4}
2	HMDB0006218	(9cis)-Retinal	C20H28O	1.477	1.02×10^{-3}
3	HMDB0005048	10(E), 12(Z)-Conjugated linoleic acid	C18H32O2	1.124	1.66×10^{-4}
4	HMDB0032090	12-oxo Phytodienoic Acid	C18H28O3	1.149	3.78×10^{-5}
5	HMDB0006294	16-Hydroxyhexadecanoic acid	C16H32O3	1.695	1.22×10^{-4}
6	HMDB0011568	1-Linoleoyl glycerol	C21H38O4	1.434	7.40×10^{-5}
7	HMDB0011564	1-Palmitoylglycerol	C19H38O4	1.021	2.92×10^{-5}
8	HMDB0011131	1-Stearoylglycerol	C21H42O4	1.000	2.11×10^{-2}
9	HMDB0059709	2-Hydroxybenzyl alcohol	C7H8O2	2.268	5.54×10^{-5}
10	HMDB01624	2-Hydroxycaproic acid	C6H12O3	1.780	6.15×10^{-6}
11	HMDB0003540	3'-Adenosine monophosphate (3'-AMP)	C10H14N5O7P	1.206	2.95×10^{-2}
12	HMDB0000779	3-Phenyllactic acid	C9H10O3	1.573	3.78×10^{-6}
13	HMDB0001173	5'-S-Methyl-5'-thioadenosine	C11H15N5O3S	1.146	2.72×10^{-2}
14	HMDB0012273	Adenine	C5H5N5	1.289	2.36×10^{-3}
15	HMDB0000045	Adenosine 5'-monophosphate	C10H14N5O7P	1.221	5.29×10^{-3}
16	HMDB0028699	Alanyltyrosine	C12H16N2O4	1.301	3.06×10^{-4}
17	HMDB0001043	Arachidonic acid	C20H32O2	1.307	4.10×10^{-5}
18	HMDB0000168	L-Asparagine	C4H8N2O3	1.050	1.12×10^{-3}
19	HMDB0001870	Benzoic acid	C7H6O2	1.011	1.07×10^{-3}
20	HMDB0000097	Choline	C5H14NO	1.075	3.35×10^{-3}
21	HMDB0000641	L-Glutamine	C5H10N2O3	1.203	1.17×10^{-2}
22	HMDB0000163	Maltose	C12H22O11	1.209	2.79×10^{-5}
23	HMDB0000651	Decanoylcarnitine	C17H33NO4	1.042	1.61×10^{-3}
24	HMDB0000929	L-Tryptophan	C11H12N2O2	1.124	3.94×10^{-2}
25	HMDB0003213	Raffinose	C18H32O16	1.177	7.03×10^{-3}
26	HMDB0000606	D-α-Hydroxyglutaric acid	C5H8O5	1.400	2.11×10^{-3}
27	HMDB0001999	Eicosapentaenoic acid	C20H30O2	1.748	9.72×10^{-7}
28	HMDB0000573	Elaidic acid	C18H34O2	1.397	1.06×10^{-4}
29	HMDB0034153	Ethyl myristate	C16H32O2	1.223	1.78×10^{-4}
30	HMDB0004472	Eucalyptol	C10H18O	1.343	2.27×10^{-3}
31	HMDB0000625	Gluconic acid	C6H12O7	1.729	3.04×10^{-2}
32	HMDB0000132	Guanine	C5H5N5O	1.405	1.23×10^{-3}
33	HMDB0001397	Guanosine monophosphate (GMP)	C10H14N5O8P	1.126	2.61×10^{-2}
34	HMDB0000130	Homogentisic acid	C8H8O4	1.897	1.33×10^{-2}
35	HMDB0014613	Isoferulic acid	C10H10O4	1.225	4.59×10^{-5}
36	HMDB0000191	Aspartic acid	C4H7NO4	1.462	5.38×10^{-4}
37	HMDB0011175	Leucylproline	C11H20N2O3	1.072	4.68×10^{-3}
38	HMDB0000125	Glutathione (reduced)	C10H17N3O6S	1.518	1.85×10^{-4}
39	HMDB0000943	Threonic acid	C4H8O5	2.710	4.05×10^{-7}
40	HMDB0000167	L-Threonine	C4H9NO3	1.007	3.92×10^{-2}
41	HMDB0000691	Malonic acid	C3H4O4	1.694	2.17×10^{-5}
42	HMDB0000512	N-Acetyl-L-phenylalanine	C11H13NO3	1.170	8.96×10^{-5}
43	HMDB0001488	Nicotinic acid	C6H5NO2	1.742	2.17×10^{-5}
44	HMDB0002117	Oleamide	C18H35NO	1.646	1.49×10^{-5}
45	HMDB0002364	Oleanolic acid	C30H48O3	1.748	9.12×10^{-5}
46	HMDB0003229	Palmitoleic acid	C16H30O2	1.204	2.34×10^{-4}
47	HMDB0003689	Protectin D1	C22H32O4	1.405	3.99×10^{-4}
48	HMDB0000252	Sphingosine (d18:1)	C18H37NO2	1.062	5.93×10^{-3}
49	HMDB0000300	Uracil	C4H4N2O2	1.102	6.54×10^{-3}
50	HMDB0000288	Uridine monophosphate (UMP)	C9H13N2O9P	1.028	2.62×10^{-2}

51	HMDB0032012	Vanillyl alcohol	C8H10O3	1.853	4.67×10^{-2}
52	HMDB0000292	Xanthine	C5H4N4O2	1.101	6.40×10^{-4}
53	HMDB0030963	α-Eleostearic acid	C18H30O2	1.030	5.72×10^{-4}
54	HMDB0013624	α-Linolenoyl ethanolamide	C20H35NO2	1.355	1.41×10^{-5}
